# Prediction of successful de-cannulation of tracheostomised patients in medical intensive care units

**DOI:** 10.1186/s12931-021-01732-w

**Published:** 2021-04-28

**Authors:** Chul Park, Ryoung-Eun Ko, Jinhee Jung, Soo Jin Na, Kyeongman Jeon

**Affiliations:** 1 Department of Critical Care Medicine, Samsung Medical Center, Sungkyunkwan University School of Medicine, 81 Irwon-ro, Gangnam-gu, Seoul, 06351 Republic of Korea; 2 Department of Pulmonary Medicine, Wonkwang Medical Center, Iksan, Republic of Korea; 3 Intensive Care Unit Nursing Department, Samsung Medical Center, Sungkyunkwan University School of Medicine, Seoul, Republic of Korea; 4 Division of Pulmonary and Critical Care Medicine, Department of Medicine, Samsung Medical Center, Sungkyunkwan University School of Medicine, 81 Irwon-ro, Gangnam-gu, Seoul, 06351 Republic of Korea

**Keywords:** Tracheostomy, Device removal, Artificial respiration, Intensive care unit, Predictive value of tests

## Abstract

**Background:**

Limited data are available on practical predictors of successful de-cannulation among the patients who undergo tracheostomies. We evaluated factors associated with failed de-cannulations to develop a prediction model that could be easily be used at the time of weaning from MV.

**Methods:**

In a retrospective cohort of 346 tracheostomised patients managed by a standardized de-cannulation program, multivariable logistic regression analysis identified variables that were independently associated with failed de-cannulation. Based on the logistic regression analysis, the new predictive scoring system for successful de-cannulation, referred to as the DECAN score, was developed and then internally validated.

**Results:**

The model included age > 67 years, body mass index < 22 kg/m^2^, underlying malignancy, non-respiratory causes of mechanical ventilation (MV), presence of neurologic disease, vasopressor requirement, and presence of post-tracheostomy pneumonia, presence of delirium. The DECAN score was associated with good calibration (goodness-of-fit, 0.6477) and discrimination outcomes (area under the receiver operating characteristic curve 0.890, 95% CI 0.853–0.921). The optimal cut-off point for the DECAN score for the prediction of the successful de-cannulation was ≤ 5 points, and was associated with the specificities of 84.6% (95% CI 77.7–90.0) and sensitivities of 80.2% (95% CI 73.9–85.5).

**Conclusions:**

The DECAN score for tracheostomised patients who are successfully weaned from prolonged MV can be computed at the time of weaning to assess the probability of de-cannulation based on readily available variables.

## Background

Tracheostomy is a common procedure performed in intensive care unit (ICU) patients who require prolonged mechanical ventilation (MV) in cases associated with acute respiratory failure and other airway issues [1, 2]. Furthermore, the development of less invasive surgical techniques, including percutaneous dilatation tracheostomies (PDTs), allowed the safe completion of the tracheostomy procedure at the patient’s bedside [2]. The numbers of conducted tracheostomies is growing because of the increased number of patients who require prolonged MV or who have difficulties in weaning from MV in medical ICUs [2–4]. There are various benefits associated with this procedure, including the improvement of patient comfort, a reduced need for sedation, and a lowered airway resistance, that allow an easier airway care [1, 2]. However, the presence of a tracheostomy tube in the trachea can cause complications, including tracheal stenosis, bleeding, infection, and fistula formation [5]. Therefore, the removal of tracheostomy tubes should be considered when possible complications associated with the tube placement have been resolved [6].

The process of the tracheostomy tube removal, known as de-cannulation is an important step in the recovery from chronic critical illness, and should be started when MV is no longer needed [7]. However, patients with tracheostomy tube are susceptible to muscle fatigue and to other causes of respiratory difficulty, as well as to other complications related to the tracheostomy tube itself [6, 7]. Old age, prolonged MV support, and muscle weakness have been reported as risk factors for de-cannulation failure [8, 9]. In addition, excess concern on de-cannulation failure among patients with such vulnerabilities tends to delay the assessment of readiness for capping trial or de-cannulation. Therefore, it is necessary to predict at the time of weaning from MV whether de-cannulation is possible or additional interventions is required for successful de-cannulation.

Despite the relevance and importance of de-cannulation, there is no universally accepted protocol for its performance [7, 10–12]. In addition, several observational studies have shown factors associated with successful de-cannulation [9, 13–17]. However, a problem with these predictors is related to the fact that cannot be characterized easily in clinical practice because they either require specific instruments (such as the cough peak flow meter) or cannot be quantified (for example, qualitative indices such as “enough” or “high” peripheral muscle strength are used instead). Therefore, we conducted a retrospective observational study with prospectively registered data to evaluate factors associated with failed de-cannulations to develop a prediction model that could be easily be used at the time of weaning from MV.

## Methods

### Study population

**Fig. 1:**
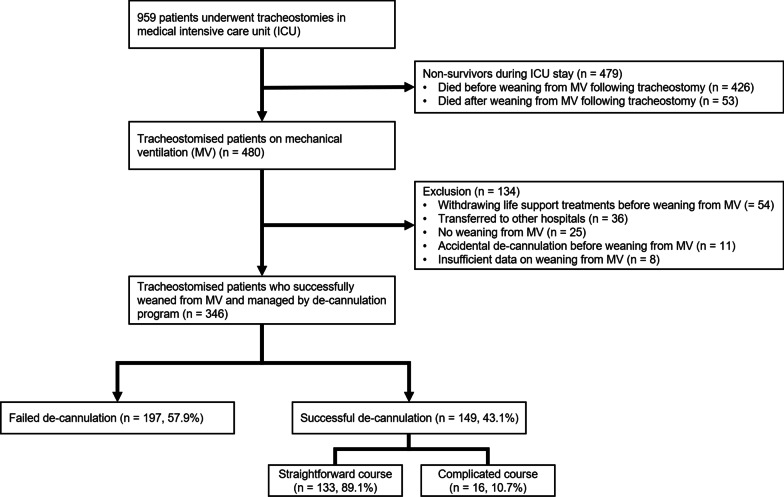
Patient flow diagram

All consecutive adult patients aged 18 or older who underwent tracheostomy were prospectively registered at the Samsung Medical Center (a 1,989-bed, university-affiliated, tertiary referral hospital in Seoul, South Korea) [18]. During the study period of 5 years, a total of 959 consecutive patients underwent tracheostomy, which were performed at the patient bedside using bronchoscopy-guided PDT techniques (Cook Medical Inc., Bloomington, IN, USA) in medical ICUs. To address the primary research objective associated with the determination of the factors related with failed de-cannulation, patients who expired in ICU (n = 479), whose life support treatments were withdrawn before weaning from MV (n = 54), who were transferred to other hospitals (n = 36), who were not weaned from MV (n = 25), accidental de-cannulation before weaning from MV (n = 11), and insufficient data on weaning from MV (n = 8) were excluded. Finally, a total of 346 adult patients who successfully weaned from MV and were managed by respiratory care practitioners (RCP) for de-cannulation according to our institutional protocol were retrieved for analysis (Fig. 1).

The institutional review board of the Samsung Medical Center approved this study and waived the requirement for informed consent because of the observational nature of the research. Additionally, the patients’ information was anonymized and eliminated from records or files prior to analysis.

### Standardized de-cannulation program

**Fig. 2:**
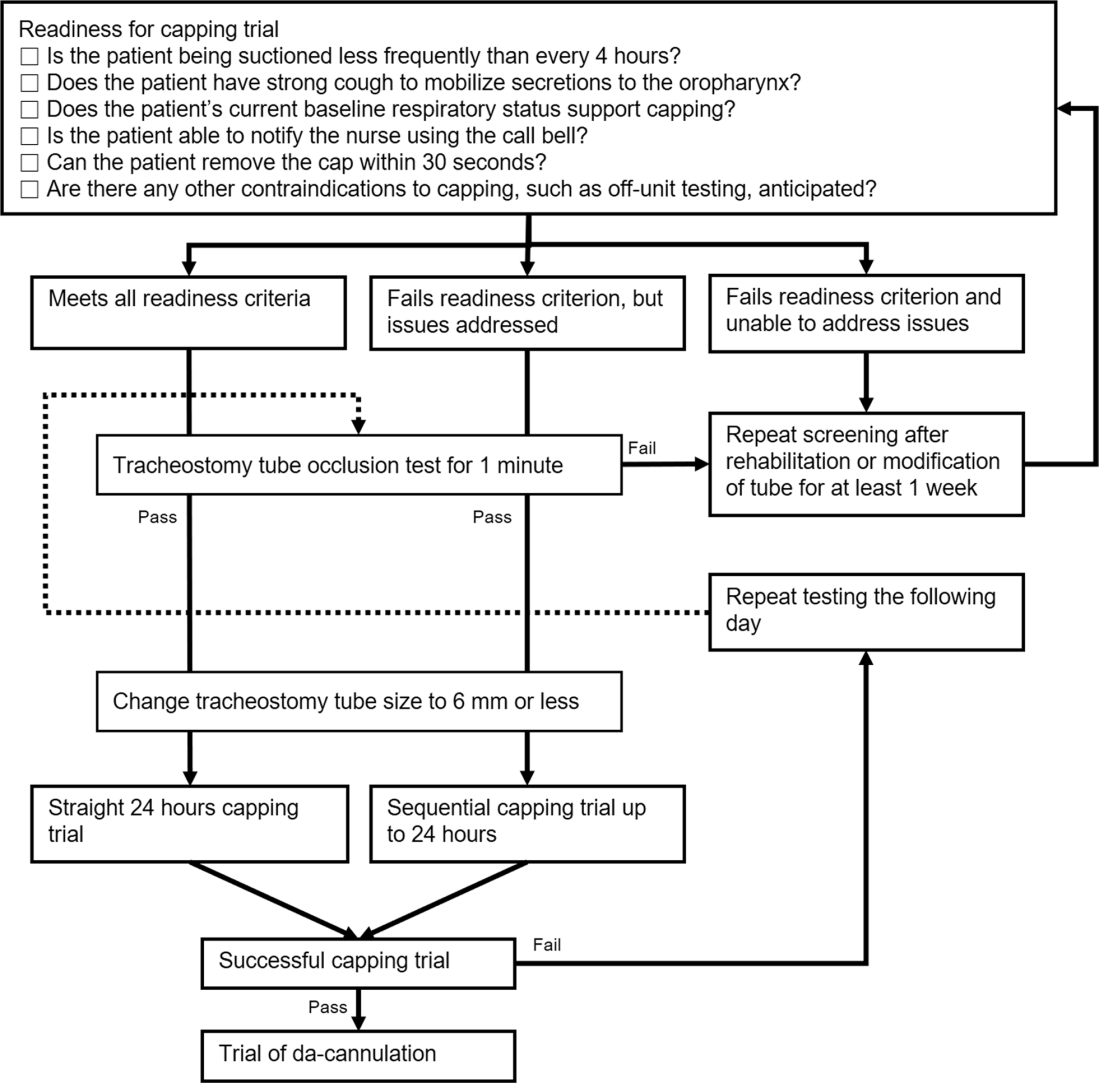
Standardized tracheostomy tube capping and de-cannulation algorithm

Our hospital implemented a standardized de-cannulation program at the beginning of June 2014. The attending physicians who cared for patients with a tracheostomy identified potential adult candidates for the de-cannulation requested evaluation for de-cannulation when the underlying indication for tracheostomy had resolved. The RCP, who are registered nurses specialized in respiratory care, screened for readiness for capping trials based on the tracheostomy capping checklist formulated and published previously [7, 19]. The checklist consisted of the volume of tracheal secretion, frequency of tracheal suction, cough effectiveness, and tracheostomy tube occlusion test (Fig. 2). The intact upper airway was evaluated with the tracheostomy tube occlusion test. Specifically, the noninvasive evaluation involved the full deflation of the cuff on the tracheostomy tube and the placement of a gloved finger over the tube to deflect the presence of air through the upper airway and vocal cords to allow phonation. If the patient was unable to phonate, had stridor or laboured breathing, or manifested any respiratory distress, the patient was referred to an otolaryngologist for endoscopic examination of his or her airways. Patients who passed the screening criteria were challenged with a 24 h capping trial. If the criteria were not initially met but subsequently addressed, a more conservative approach was adopted in which the patient was capped initially for 12 h and then uncapped for 12 h before proceeding to the 24 h capping trial. If the patient did not meet the criteria, modifications including downsizing of the tracheostomy tube and rehabilitation were made to allow the capping trial to proceed safely. For example, if the tracheostomy tube was size ≥ 6.0, then the tube was changed to the smaller one by 1.0 or 2.0. Physical rehabilitation was provided in patients with muscle weakness, especially who were unable to remove the cap or notify the nurse using their call bell, according to individual treatment plans, such as regaining walking function and activities of daily living.

Capping was defined as successful if there was no oxygen desaturation, if the oxygen requirement had not increased to 40% FiO_2_ or higher, or if the cap or plug had not been removed for any reason, such as suctioning, desaturation, shortness of breath, or hemodynamic instability. If the patient did not tolerate capping, the trial was aborted, the RCP and attending physician were notified, and the capping trial was restarted the following day using the conservative approach discussed above. If the patient passed the capping trial, the result was shared with attending physician for decision whether to proceed de-cannulation. The tracheostomy tube was removed and the stoma was covered with a sterile gauze ball and Primapore dressing. Following de-cannulation, the patients were monitored with continuous pulse oximetry for at least 24 h.

### Data collection and clinical outcomes

Over the study period, all patients who underwent tracheostomy in the ICU were prospectively registered. The following information was collected on each registered patient: demographic data, underlying conditions, laboratory data, severity scores—including the simplified acute physiology score (SAPS) 3 [20] and the sequential organ failure assessment (SOFA) score [21]—reasons for MV, and data on tracheostomy, including reasons for tracheostomy, timing of the procedure, and complications developed during the procedure or within 24 h after the procedure. Additional clinical data at the time of successful weaning from MV were collected by a retrospective review of electronic hospital records: Charlson co-morbidity index (CCI) [22], SOFA score, presence of neurologic disease, presence of delirium assessed by the confusion assessment method for the ICU (CAM–ICU) [23], development of pneumonia following tracheostomy, frequency of endotracheal suction, requirement of vasoactive agents and renal replacement therapy, and physiologic and laboratory parameters.

The indication of tracheostomy was classified according to the following four categories: (1) prolonged ventilation, defined as MV support over 7 days, (2) predicted to be difficult-to wean, patients who had expected to prolonged MV support or repeatedly failed to liberation from MV support within 7 days of MV support, (3) reduced level of consciousness, patients who had irreversible or deteriorated neurological disorder, need for prolonged intra-tracheal suction, dysphagia with risk of aspiration, or had incompetence caused by critical illness, (4) upper airway obstruction, patients who had anatomical abnormalities, including tumours, bilateral vocal cord paralysis, tracheomalacia, or post-intubation tracheal stenosis. Successful de-cannulation was defined as the state in which there were no respiratory complications until hospital discharge. Failed de-cannulation was defined as the state in which patients still had a tracheostomy tube at the time of their hospital discharge owing to recurrent capping trial failures until hospital discharge or re-insertion of the tracheostomy tube before hospital discharge.

### Statistical analyses

The data are presented as medians and interquartile ranges (IQR) for continuous variables and as numbers and percentages for categorical variables. The data were analysed using Kolmogorov–Smirnov tests for normal distribution. The baseline characteristics and outcome measures of interest were compared between successful de-cannulation and failed de-cannulation groups using the Mann–Whitney U test for continuous variables and the Pearson’s chi-square test or Fisher exact test for categorical variables.

Logistic regression analysis was performed to estimate the odds ratios (ORs) of each variable and identify associated factors for the prediction of successful de-cannulation at the time of weaning from MV. The ORs of each variable are reported with their 95% confidence intervals (CIs). Variables that appeared to be related in the initial analysis with a *P* value of less than 0.2 were considered in the multivariable regression model [24]. To reduce the risk of multi-collinearity, closely correlated variables were candidates for inclusion in the final model. The goodness-of-fit of the model was evaluated with the Hosmer–Lemeshow test.

From the logistic regression findings, a score-based predictive scoring system was developed [25]. Continuous variables were entered after their conversion to categorical variables to facilitate clinical interpretation. To generate a simple integer-based point score for each predictor variable, we assigned scores by dividing the β coefficients by the absolute value of the smallest coefficient in the final model and rounded up to the nearest integer. The total score for each participant was calculated by adding each component together. We then explored the predictive value of the point score for correctly indicating the failed de-cannulation using a receiver operating characteristic (ROC) curve and assessed both the discrimination (via the C index) and calibration (using Hosmer–Lemeshow statistics) of the score. In addition, the method of leave-one-out cross-validation (LOOCV) was used for internal validation to obtain the mis-classification error rate [26]. Statistical analyses were performed with STATA® (version 12, STATA Corporation, College Station, TX, USA). All tests were two-tailed, and *P* < 0.05 was considered statistically significant.

## Results

Among the 346 adult tracheotomised patients who successfully weaned from MV and managed by the de-cannulation program, 149 (43.1%) patients were successfully de-cannulated during the same hospitalization period (Fig. 1). Of 149 with successful de-cannulation, 133 (89.3%) patients had a straightforward course and 16 (10.7%) patients had a complicated course. No patient required the re-insertion of the tracheostomy tube after de-cannulation. The baseline characteristics of patients are listed in Table 1. There were 231 males, and the median age was 62.8 (IQR, 47.0–78.6) years. The median SAPS 3 and SOFA scores on ICU admission were 53.9 (39.0–68.8) and 7.6 (3.4–11.8), respectively. Pneumonia was the most common cause of MV support (n = 138, 39.9%), followed by extra-pulmonary sepsis (n = 62, 17.9%) and coma (n = 55, 15.9%). The most common reason for tracheostomy was prolonged ventilation (n = 196, 50.9%) followed by predicted to be difficult-to wean (n = 115, 33.2%). The median time from intubation to tracheostomy was 9.1 (2.4–15.8) days.

**Table 1:** Baseline characteristics at the time of tracheostomy in patients who underwent this procedure in medical intensive care units

Characteristics	No. of patients (%) or median (IQR)
Age, years	62.8 (47.0–78.6)
Male	231 (66.8)
BMI, kg/m^2^	22.2 (18.1–26.3)
Underlying disease Malignant disease Respiratory disease Neurologic disease Genitourinary disease Cardiovascular disease	135 (39.0) 42 (12.1) 72 (20.8) 33 (9.5) 52 (15.0)
Charlson co-morbidity index	4.5 (1.8–7.2)
Severity score at ICU admission SAPS3 SOFA	53.9 (39.0–68.8) 7.6 (3.4–11.8)
GCS	9.2 (4.4–14.0)
Cause of MV support Pneumonia Extra-pulmonary sepsis Coma Pulmonary oedema ARDS Exacerbation of ILD Post CPR Central airway obstruction	138 (39.9) 62 (17.9) 55 (15.9) 17 (4.9) 27 (7.8) 5 (1.4) 25 (7.2) 17 (4.9)
Indication of tracheostomy Prolonged ventilation Predicted to be difficult-to wean Reduced level of consciousness Upper airway obstruction	176 (50.9) 115 (33.2) 110 (31.8) 15 (4.3)
Time from intubation to tracheostomy, days	9.1 (2.4–15.8)
Tracheostomy related adverse events	32 (9.2)

*No.* number, *IQR* interquartile range, *BMI* body mass index, *ICU* intensive care unit, *SAPS* simplified acute physiology score, *SOFA* sequential organ failure assessment, *GCS* Glasgow coma scale, *MV* mechanical ventilation, ARDS acute respiratory distress syndrome, *ILD* interstitial lung disease, *CPR* cardiopulmonary resuscitation

Uni-variate comparisons of the clinical characteristics at the time of weaning from MV between successful and failed de-cannulation groups are presented in Table 2. The failed de-cannulation group had an increased co-morbidity, including neurologic disease and a worse organ dysfunction than the successful de-cannulation group, although there was no difference in the duration of MV. Delirium assessed by CAM–ICU and endotracheal suction frequency was higher in the failed de-cannulation group than in the successful de-cannulation group. The need for vasopressor at the time of weaning from MV, and the prevalence of pneumonia during MV following tracheostomy, were also greater in the failed de-cannulation group than in the successful de-cannulation group. In addition, better oxygenation at the time of weaning from MV was associated with successful de-cannulation. However, the need for renal replacement therapy was similar between the two groups.

**Table 2:** Univariate comparisons of clinical characteristics at the time of weaning from mechanical ventilation between successful and failed de-cannulation groups

Characteristics	Successful de-cannulation (n = 149)	Failed de-cannulation (n = 197)	*P* value
Age, years	57.9 (41.0–74.8)	66.5 (52.6–80.4)	< 0.001
Male	93 (62.4%)	138 (70.1%)	0.168
BMI, kg/m^2^	23.0 (19.0–27.0)	21.6 (17.5–25.7)	0.002
Underlying disease Malignant disease Respiratory disease Neurologic disease Genitourinary disease Cardiovascular disease	48 (32.2%) 22 (14.8%) 18 (12.1%) 19 (12.8%) 22 (14.8%)	87 (44.2%) 20 (10.2%) 54 (27.4%) 14 (7.1%) 30 (15.2%)	0.032 0.256 0.001 0.113 > 0.999
Charlson co-morbidity index	3.9 (1.1–6.7)	5.0 (2.4–7.6)	< 0.001
Cause of MV support Pneumonia Extra-pulmonary sepsis Coma Pulmonary oedema ARDS Exacerbation of ILD Post CPR Central airway obstruction	70 (47.0%) 24 (16.1%) 9 (6.0%) 10 (6.7%) 19 (12.8%) 4 (2.7%) 7 (4.7%) 6 (4.0%)	68 (34.5%) 38 (19.3%) 46 (23.4%) 7 (3.6%) 8 (4.1%) 1 (0.5%) 18 (9.1%) 11 (5.6%)	0.026 0.534 < 0.001 0.274 0.005 0.220 0.171 0.680
Duration of MV, days	21.9 (2.2–39.6)	18.1 (0.3–38.0)	0.073
Charlson co-morbidity index	3.9 (1.1–6.7)	5.0 (2.4–7.6)	< 0.001
SOFA score	2.7 (0.8–4.6)	4.6 (2.0–7.2)	< 0.001
Positive CAM–ICU	48 (32.2%)	159 (80.7%)	< 0.001
Change of GCS Decreased No change Increased	18 (12.1%) 56 (37.6%) 75 (50.3%)	54 (27.4%) 71 (36.0%) 71 (36.0%)	0.001 0.855 0.011
Suction frequency within 24 h	8.0 (8.0–10.0)	14.0 (12.0–16.0)	< 0.001
Weaning index Respiratory rate, /min PaO_2_/FiO_2_ ratio RSBI	20.0 (16.0–23.0) 350.0 (286.0–411.0) 43.0 (32.0–55.0)	19.0 (16.0–24.0) 311.0 (250.0–382.0) 49.0 (32.0–65.0)	0.848 0.001 0.059
Laboratory findings Total bilirubin, mg/dL Creatinine, mg/dL Lactic acid, mmol/L	0.9 (0.5–1.7) 0.9 (0.6–1.4) 1.1 (0.7–1.5)	0.6 (0.4–1.1) 0.7 (0.5–1.1) 1.7 (0.7–2.7)	< 0.001 0.009 < 0.001
Vasopressor requirement	5 (3.4%)	28 (14.2%)	0.001
CRRT	4 (2.7%)	7 (3.6%)	0.883
Post-tracheostomy pneumonia during MV	36 (24.2%)	88 (44.7%)	< 0.001

Data are presented as number (percentage) or as median (interquartile range)

*BMI* body mass index, *MV* mechanical ventilation, ARDS, acute respiratory distress syndrome, *ILD* interstitial lung disease, *CPR* cardiopulmonary resuscitation, *SOFA* sequential organ failure assessment; CAM–ICU, confusion assessment method for the intensive care unit, *GCS* Glasgow coma scale, *PaO*_*2*_ partial pressure of oxygen, *FiO*_*2*_ fraction of inspired oxygen, *RSBI* rapid shallow breathing index, *CRRT* continuous renal replacement therapy

The results of uni-variable and multi-variable analyses with the logistic regression model are presented in Table 3. Logistic regression analysis identified 11 variables that were independently associated with failed de-cannulation: older age, lower body mass index (BMI), higher suction frequencies within 24 h before MV weaning, lower PaO_2_/FiO_2_ ratio, malignancy as one of the co-morbidities, non-respiratory causes of MV including extra-pulmonary sepsis, coma, and post-cardiac arrest, presence of neurologic disease, delirium assessed by CAM–ICU, vasopressor requirement at the day of weaning from MV, and presence of post-tracheostomy pneumonia, were independently associated with failed de-cannulation (Table 3).

**Table 3:** Uni-variable and multi-variable analyses with logistic regression models for variables associated with failed de-cannulation in tracheotomised patients who succeeded to weaning from mechanical ventilation

Variables	Uni-variable			Multi-variable		
	Crude OR	95% CI	*P*	Adjusted OR	95% CI	*P*
Age, years	1.04	1.02–1.05	< 0.001	1.04	1.01–1.07	0.019
BMI, kg/m^2^	0.92	0.87–0.97	0.002	0.82	0.73–0.92	0.001
Suction frequency, per day	1.65	1.50–1.82	< 0.001	1.82	1.55–2.20	< 0.001
PaO_2_/FiO_2_ ratio	1.00	0.99–1.00	0.001	0.99	0.99–1.00	0.002
Lactic acid	5.75	3.50–10.03	< 0.001	9.31	3.87–26.35	< 0.001
Underlying malignancy	1.66	1.07–2.61	0.025	5.37	2.04–15.53	0.001
Non-respiratory causes of MV support	2.99	1.90–4.75	< 0.001	5.11	1.84–15.19	0.002
Neurologic disease at weaning from MV	6.05	3.81–9.77	< 0.001	5.11	2.06–13.58	< 0.001
Delirium at weaning from MV	8.80	5.43–14.57	< 0.001	11.13	4.40–31.50	< 0.001
Vasopressor at weaning from MV	4.77	1.95–14.33	0.002	6.89	1.23–51.27	0.039
Post-tracheostomy pneumonia before weaning from MV	2.53	1.60–4.08	< 0.001	5.45	2.04–16.02	0.001

*OR* adds ratio, *CI* confidence interval, *BMI* body mass index, *MV* mechanical ventilation

**Fig. 3:**
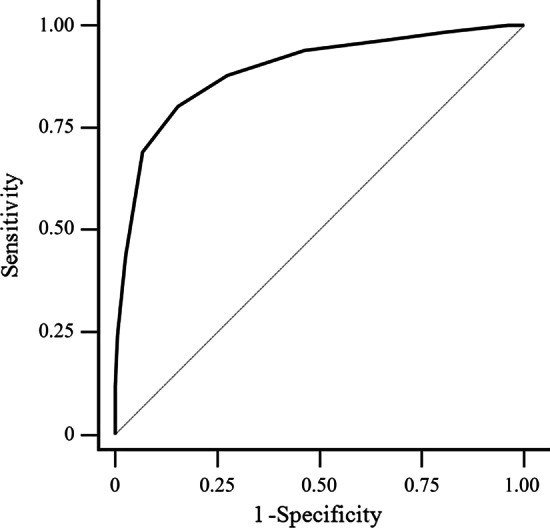
Receiver operating characteristic curve of predicting failed de-cannulation with the DECAN score. The area under the curve is 0.890 (95% confidence interval, 0.853–0.921)

Based on the logistic regression analysis, we created a new predictive scoring system, referred to as the DECAN score, using the β coefficients (Table 4). For the convenient utility, we excluded several items in tracheostomy capping checklist as follows: suction frequencies and PaO_2_/FiO_2_ ratio, and converted the continuous variables to categorical variables with ROC curve analysis for optimal cut-off values as follows: age > 67 years and BMI < 22 kg/m^2^. Serum lactic acid level also excluded due to the usual optimal cut-off value of 1.3 mmol/L, with only 5 patients exceeding 4 mmol/L. A β coefficient of 1.0 corresponded to approximately 1 point. Therefore, points were assigned as follows: 1 point for age > 67 years (β = 1.25), BMI < 22 kg/m^2^ (β = 1), underlying malignancy (β = 1.02), and non-respiratory causes of MV (β = 1.24); 2 points for presence of neurologic disease (β = 2.18), vasopressor requirement (β = 1.86), and presence of post-tracheostomy pneumonia (β = 1.54); 3 points for presence of delirium (β = 2.97) that resulted in a maximum of 13 possible points for the DECAN score to predict the probability of failed de-cannulation. The DECAN score exhibited good calibration and discrimination characteristics, with a goodness-of-fit of 0.6477 and an area under the ROC curve of 0.890 (95% CI, 0.853–0.921) (Fig. 3). LOOCV analysis of the score for internal validation showed that it had a low-misclassification error rate of failed de-cannulation at 9.5%. The optimal cut-off DECAN score used for the prediction of successful de-cannulation was ≤ 5 points. This was associated with specificities of 84.6% (95% CI 77.7–90.0) and sensitivities of 80.2% (95% CI 73.9–85.5). The positive predictive value was 83.9% and the negative predictive value was 81.0%.

**Table 4:** Prediction scoring system of factors associated with failed de-cannulation in tracheotomised patients who succeeded to weaning from mechanical ventilation

Valuables	ß coefficient	Adjusted OR	95% CI	P	Score points
Age > 67, years	1.25	3.09	2.12–7.36	< 0.001	1
BMI < 22, kg/m^2^	1	2.48	1.36–4.61	0.003	1
Underlying malignancy	1.02	2.53	1.35–4.87	0.004	1
Non-respiratory causes of MV support	1.24	3.08	1.64–5.92	< 0.001	1
Neurologic disease at weaning from MV	2.18	5.41	2.94–10.26	< 0.001	2
Delirium at weaning from MV	2.97	7.36	4.02–13.93	< 0.001	3
Vasopressor requirement at weaning from MV	1.86	4.62	1.39–18.72	0.019	2
Post-tracheostomy pneumonia	1.70	4.21	2.22–8.30	< 0.001	2

*OR* odd ratio, *CI* confidence interval, *BMI* body mass index, *MV* mechanical ventilator

## Discussion

This study evaluated the risk factors for failed de-cannulations in tracheotomised patients who were (a) successfully weaned from MV and (b) managed by a standardized de-cannulation program. It also applied a risk stratification model to identify/predict the patients who would be successfully de-cannulated at the time of weaning from MV. Age, BMI, co-morbidities, such as malignancy and neurologic disease, suction frequencies, level of lactic acid, degree of oxygenation, delirium, and vasopressor requirement on the day of weaning from MV, and pneumonia after tracheostomy, were associated with failed de-cannulation. In addition, we developed and validated the clinical score which was determined at the time of weaning from MV. The DECAN score reliably assessed the probability of successful de-cannulation with a positive predictive value of 84% for scores of ≤ 5.

The number of tracheostomies has increased and the rate at which the procedures have been conducted has also increased with the advancement of critical care [1–4]. In spite of this increase, there was no consensus on the standard approach that was used for de-cannulation [7, 10–12, 15]. Prospective, multi-centre, observational studies revealed that 54% of participants were weaned from prolonged MV with tracheostomy, but only 59% of those weaned were ultimately de-cannulated [27, 28]. Therefore, for a therapeutic optimization of tracheostomy management, it is of great interest to identify the factors that contribute to the success or failure of de-cannulation. Previous studies have shown that factors associated with successful de-cannulation included the peak cough flow and respiratory muscular strength [9, 14, 16, 17]. In addition, a systematic review showed that effective coughing and tolerance of tracheostomy tube occlusion ≥ 24 h were the most relevant parameters for de-cannulation in clinical practice. However, these predictors should be assessed at the time of tube capping trials for de-cannulation with specific measuring instruments and are difficult to quantify. A recent observational study identified practically measurable factors before the tube capping trial for de-cannulation [10]. Additionally, increasing age, prolonged duration of MV, and other complications, were negatively associated with the probability of de-cannulation, but oral diet and higher alertness at admission were positively associated. In the present study, predictors of failed de-cannulation from medical routine data could be determined in a large number of tracheotomised patients who were successfully weaned from MV. Although many of our predictors have been found to be associated with de-cannulation in previous studies, suction frequencies, high-lactic acid level, lower oxygenation, delirium, and vasopressor requirements on the day of the weaning from MV, were also significantly associated with failed de-cannulations even after successful weaning from MV. These results suggest that the assessment of a patient’s probability for de-cannulation should be considered immediately after the weaning from prolonged MV.

Even though many factors have been identified as predictors of successful de-cannulation [9, 13–17], objective quantitative variables should be taken into greater account in the decision process. Based on the results of the systematic review on the factors associated with de-cannulation [12], a systematic scoring system has been proposed with references to the objective parameters for practical use. However, this hypothetical score has never been validated in clinical practice. In a relatively large population of tracheotomised patients who successfully weaned from prolonged MV, we developed and validated a clinical score that is easily determined at the bedside, and reliably assessed the probability of successful de-cannulation. The score exhibited good discrimination and calibration. Therefore, the DECAN score reported herein may encourage clinicians to assess the probability of successful de-cannulation at the time of weaning from prolonged MV in tracheotomised patients. Moreover, with a negative predictive value of 81% for scores > 5, it is suggested that the score appears useful as a reference test for additional multidisciplinary management of tracheostomised patients to promote de-cannulation after successful weaning from MV, although final decision whether to proceed de-cannulation was made by attending physician based on the readiness criteria and results of capping trial, not the DECAN score.

To evaluate tracheotomised patients for de-cannulation, it is imperative that the predictors for de-cannulation readiness and criteria for capping be identified to minimize the risk of respiratory compromise. Recently, a multi-disciplinary protocol used for the determination of the readiness of tracheotomised patients for a trial of capping prior to de-cannulation has been proposed. This protocol has been associated with a significant decrease in adverse events associated with tracheostomy de-cannulation [19]. In addition, the multi-disciplinary standardized capping and de-cannulation protocol provided a high rate of successful de-cannulation when the screening tool and algorithm were appropriately applied. In this study, we also evaluated the de-cannulation readiness using the tracheostomy capping checklist based on previous reports [7, 19]. This has possibly contributed to the increased rate of successful de-cannulations in our cohort that may have influenced the significance of our results.

There are several limitations associated with our study. First, given its observational nature, there could be a selection bias that possibly influenced the significance of our findings. However, the data were collected prospectively from all consecutive patients who were admitted to the medical ICU and tracheotomised. The patients were managed by RCP for de-cannulation according to our institutional protocol. Second, the present study was conducted at a single institution with a standardized de-cannulation program. Accordingly, our findings may have limited generalizability and additional external validations with larger samples are warranted to implement the score in real practice. Third, we evaluated the association of the final de-cannulation outcome with clinical data only on the day of weaning from prolonged MV. Although all patients enrolled in this study were managed with a standardized program for screening for de-cannulation, data related to changes of the clinical condition and management of the tracheotomised patients before the screening for capping trials could not be collected. Therefore, our results cannot be compared directly with previous data for the identification of the predictors of de-cannulation.

## Conclusion

In summary, this study identified practically measurable predictors of de-cannulation outcome that could be used at the time of weaning from prolonged MV. Based on the predictors, a scoring system was developed and validated to assess the probability of successful de-cannulation in patients with tracheostomy. However, studies are warranted to further assess the performance of the score.

## Data Availability

The data that support the findings of this study are available on request from the corresponding author. The data are not publicly available due to privacy or ethical restrictions.

## Abbreviations

BMI: Body mass index
CAM–ICU: Confusion assessment method for the intensive care unit
CCI: Charlson co-morbidity index
CI: Confidence interval
ICU: Intensive care unit
IQR: Interquartile ranges
LOOCV: Leave-one-out cross-validation
MV: Mechanical ventilation
OR: Odds ratio
PDT: Percutaneous dilatation tracheostomy
RCP: Respiratory care practitioners
ROC: Receiver operating characteristic
SAPS: Simplified acute physiology score
SOFA: Sequential organ failure assessment
